# Supracondylar Dome Osteotomy for Post-traumatic Cubitus Varus in Children: A Retrospective Study

**DOI:** 10.7759/cureus.86893

**Published:** 2025-06-27

**Authors:** Mir Shahid-Ul Islam, Vishal Sidhu, Naim Akbar, Altaf Hussain, Mohammed Faizullah, Kumar Sanu, Muhammad A Hamid

**Affiliations:** 1 Orthopedics, Venkateshwara Institute of Medical Sciences, Gajraula, IND; 2 Orthopedic Surgery, Venkateshwara Institute of Medical Sciences, Gajraula, IND; 3 Orthopedic Surgery, University Hospitals Birmingham, Birmingham, GBR

**Keywords:** carrying angle, cubitus varus, dome osteotomy, lateral condylar prominence, pediatric elbow, supracondylar fracture

## Abstract

Objective: Cubitus varus is a frequent sequela of pediatric supracondylar humerus fractures and presents with varus malalignment, extension, and internal rotation of the elbow. Although often regarded as a cosmetic deformity, it can lead to significant functional impairments, including chronic pain, ulnar nerve palsy, and posterolateral elbow instability.We retrospectively analyzed the effectiveness of supracondylar dome osteotomy in restoring alignment and function in children with post-traumatic cubitus varus.

Methods: Medical records of 18 children aged over five years who underwent supracondylar dome osteotomy for cubitus varus between 2022 and 2024 were reviewed and analyzed at a tertiary care center. Exclusions included non-traumatic deformity, prior ipsilateral surgery, neurovascular involvement, or incomplete data. Pre- and postoperative assessments included humerus-ulnar angle (HUA), elbow range of motion (ROM), lateral condylar prominence index (LCPI), and functional outcomes using standardized scoring systems.

Results: Mean age was 10.72±2.89 years, with predominantly (72.2%) male patients. The mean injury-to-surgery duration was 17.4±5.8 months. Most cases involved the left elbow (61.1%). Significant radiographic correction was achieved, with mean HUA improving from −12.93° ± 2.9° (varus) to 9.11° ± 2.49° (p< 0.001). Range of motion improved from a mean of 117.5° ± 11.28° flexion/9.3° ± 4.6° extension to 131.9° ± 8.0° flexion/3.68° ± 3.65° extension (p< 0.005). The LCPI normalized from −7.66% ± 6.56% to 10.13% ± 7.34% (p< 0.0001), while functional scores demonstrated a trend toward better outcomes. The majority of patients achieved excellent (83.33%) or good results (16.67%), with no reported poor outcomes.

Conclusion: Dome osteotomy is a safe and effective treatment for post-traumatic cubitus varus in children, reliably restoring alignment with excellent functional results and minimal complications.

## Introduction

Supracondylar humerus (SCH) fractures are the most common elbow fractures in children, accounting for over 60% of cases [1]. Cubitus varus deformity is the most frequent complication of these fractures, with an incidence of 10% to 57% regardless of initial treatment [2]. This triplanar deformity results from hyperextension, internal rotation, and medial rotation of the distal fragment [3]. While the primary concern is often cosmetic (gunstock deformity), long-term complications may arise due to altered elbow alignment, including reduced elbow flexion, secondary lateral condylar fractures, posterolateral instability, pain, delayed ulnar nerve palsy, and, rarely, elbow joint degeneration [4-6].

Restoring upper limb symmetry and preventing late complications has led to various corrective osteotomies. Lateral closing-wedge and step-cut osteotomies are commonly used for their simplicity in addressing coronal deformity and minimizing lateral prominence. However, cubitus varus deformities often involve hyperextension and medial rotation malalignment [6,7]. Alternative techniques include dome osteotomy, multiplanar osteotomy, and gradual correction with distraction osteogenesis [7-9]. Recently, 3D preoperative simulations and printing technology have improved deformity assessment and correction [10]. While multiplanar osteotomy, distraction osteogenesis, and 3D simulations enable three-dimensional correction, they are technically demanding and not universally accessible.

The dome-shaped osteotomy, with its rotational axis centered within the distal humeral metaphysis, effectively reduces lateral prominence while simultaneously correcting rotational deformity. Additionally, it provides excellent bone contact, ensuring stability and preserving length [3,8]. This retrospective study evaluates the clinical, functional, and radiographic outcomes of dome osteotomy for correcting post-traumatic cubitus varus deformities in pediatric patients.

## Materials and methods

Inclusion and exclusion criteria

**Figure 1:**
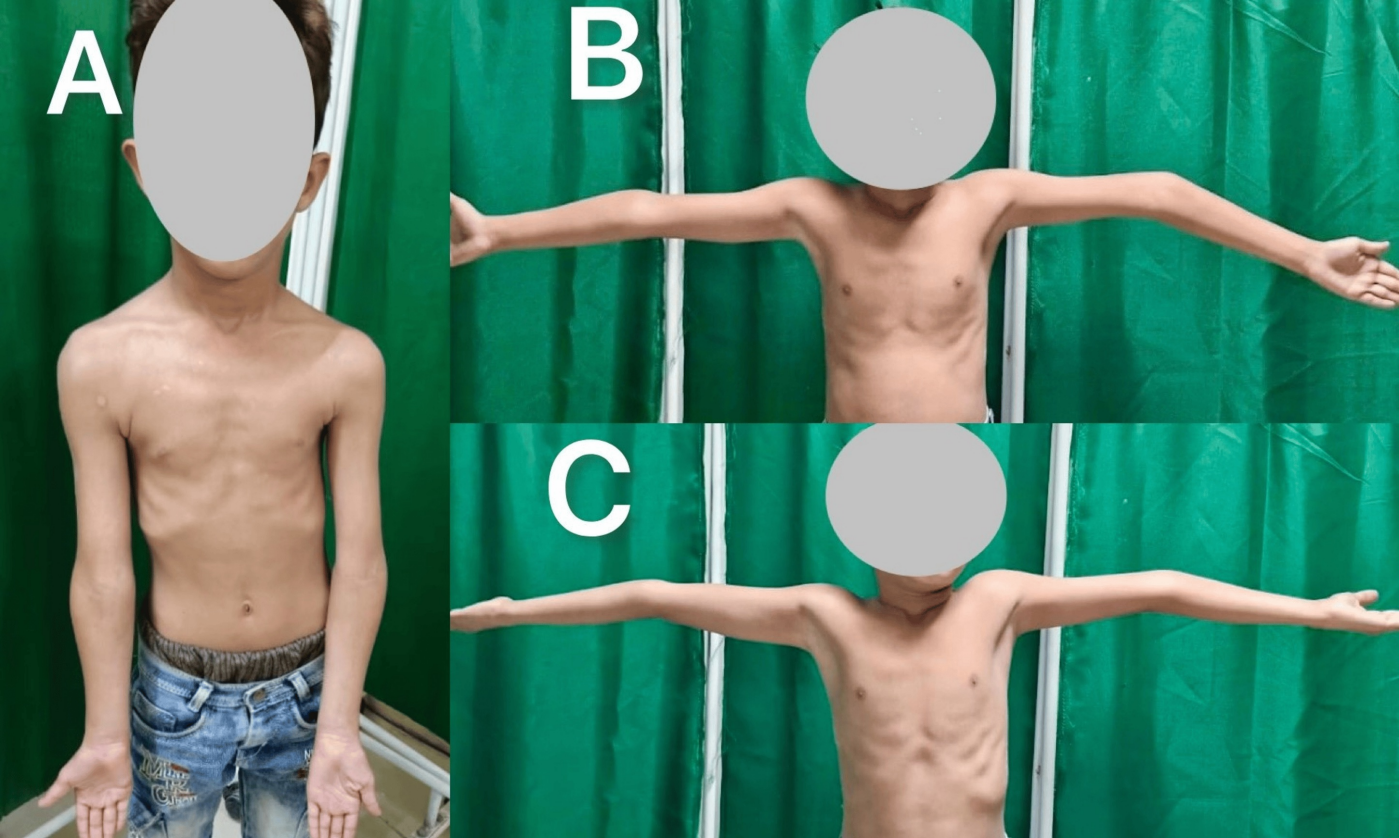
Cubitus varus secondary to a malunited SCH fracture in a child with typical deformities of varus (A & B) and hyperextension (C) SCH: Supracondylar humerus fracture

This retrospective cohort study was conducted at a tertiary care center after approval from the Institutional Ethics Committee Venkateshwara Institute of Medical Sciences in Gajraula, UP, India (approval no. VIMS/IEC/2024/94) and included children who underwent dome osteotomy for post-traumatic cubitus varus deformity between 2022 and 2024. Children aged over five years with post-traumatic cubitus varus of seven degrees or more and at least nine months duration post-trauma were included in the study (Figure 1). Patients with atraumatic varus deformity (congenital or post-septic), prior ipsilateral limb surgery, neurovascular involvement, or missing data, were excluded from the study.

Data collection

**Figure 2:**
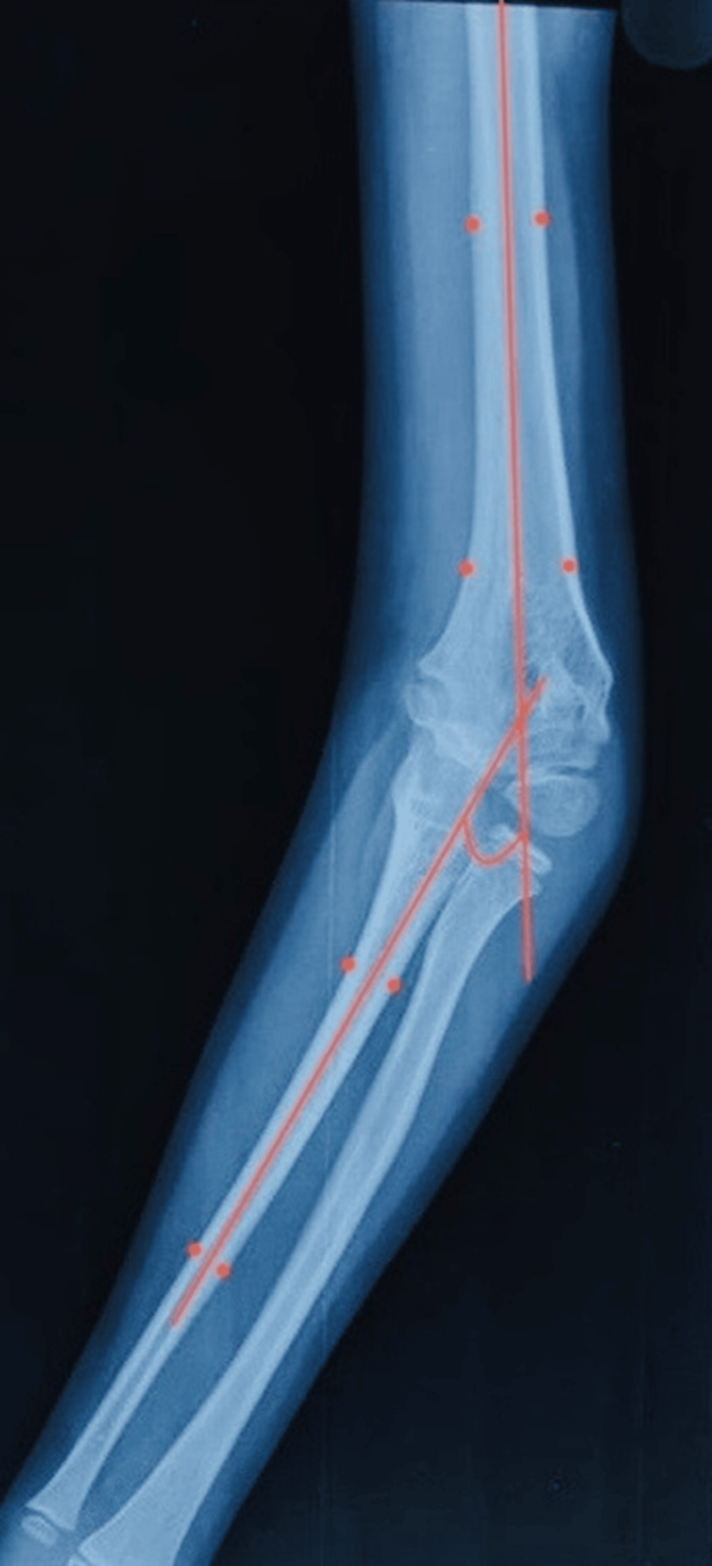
Radiograph showing HUA calculated as the angle between the mid-humeral and mid-ulnar axis HUA: Humero-ulnar angle

Patient data were systematically collected using a standardized proforma, including demographic characteristics (age at presentation, gender, affected side, and prior treatment), carrying angle (CA) measurements (both clinical and radiographic), elbow range of motion (ROM), and lateral condylar prominence index (LCPI) obtained preoperatively and postoperatively. Radiographic CA (humero-ulnar angle (HUA)) was calculated as the angle between the mid-humeral and mid-ulnar axes on preoperative and postoperative radiographs (Figure 2) [11].

**Figure 3:**
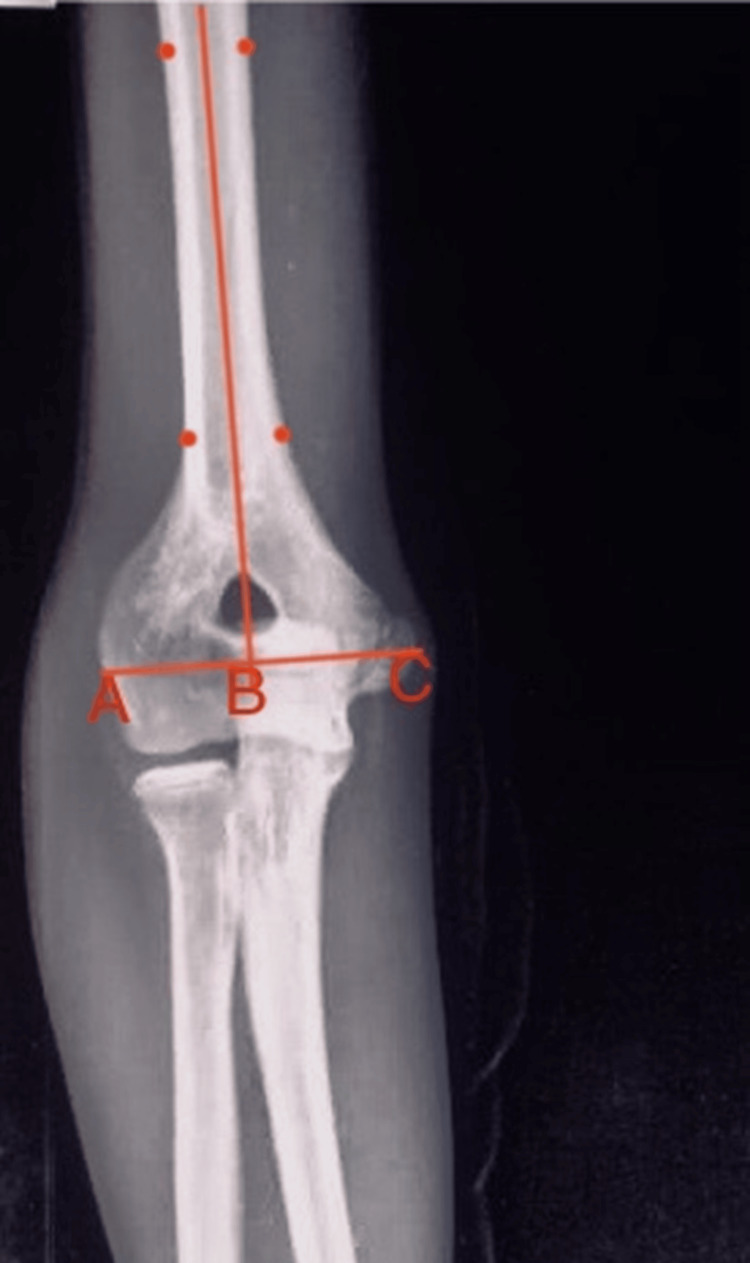
The LCPI calculated from AP elbow radiographs LCPI: Lateral condylar prominence index, AP: Anteroposterior

Clinical CA was measured using a goniometer with the forearm in supination and the elbow at neutral extension (the angle between the arm and forearm axes). The preoperative CA of the unaffected side was measured for surgical planning. Lateral condylar prominence index was calculated from anteroposterior (AP) elbow radiographs using the formula (AC - BC/AB) × 100, where point A = the most prominent point of the lateral condyle, point B = the most prominent point of the medial condyle, and point C = the intersection point of line AB with the mid-humeral line (Figure 3) [12]. To account for radiographic magnification, all measurements were standardized using the radial head diameter (adjusted to a consistent 5 mm measurement). Serial radiographs obtained during follow-up were assessed for osteotomy union and correction. Functional results were evaluated using the Mayo elbow performance score (MEPS) [13] and Banerjee criteria [14].

Surgical approach

**Figure 4:**
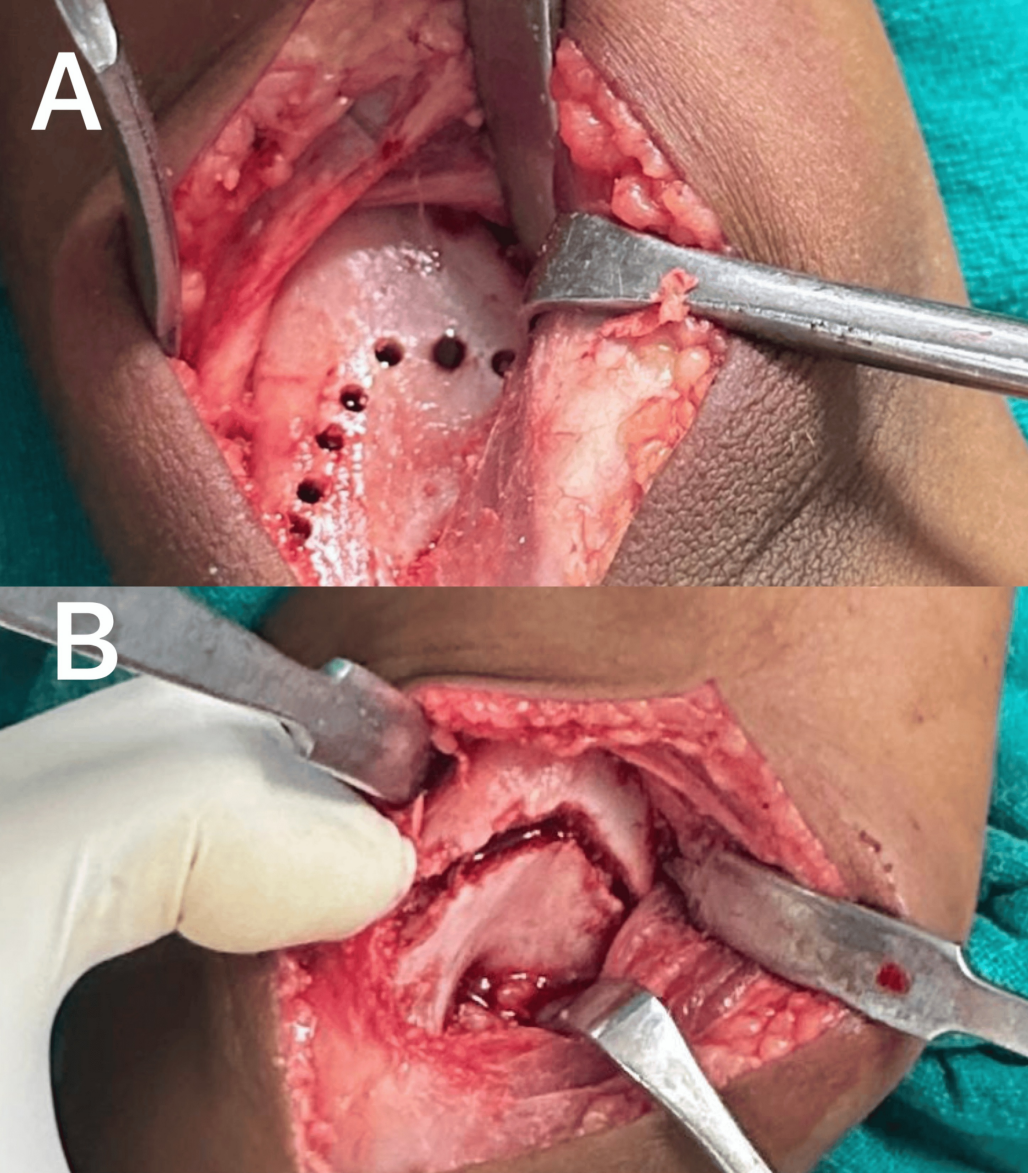
Intraoperative image showing the osteotomy site prepared with multiple drill holes (A) and completed osteotomy (B)

Patients were operated in the lateral decubitus position under general anesthesia with the affected arm supported and the forearm left free. A tourniquet was applied and inflated after administration of intravenous cefuroxime (1.5 g) 30 minutes before inflation. A reference C-arm image of the unaffected extremity was obtained for intraoperative comparison of correction. A 4 cm to 5 cm midline incision was made proximal to the olecranon tip. Full-thickness fasciocutaneous flaps were elevated, and a lateral paratricipital approach was utilized. The triceps fascia was split and mobilized from the lateral intermuscular septum. The posterior supracondylar region of the distal humerus was exposed. The dome for osteotomy was marked with a marker 1 1.5 cm proximal to the olecranon fossa. A K-wire was inserted lateral to medial (parallel to the joint line) to guide distal fragment rotation and assess correction. Multiple drill holes were made from posterior to anterior along the marked osteotomy site, and the osteotomy was completed with a small osteotome (Figure 4).

The lateral aspect of the distal fragment was freshened with an osteotome to provide a raw surface for union. The distal fragment was rotated to achieve the desired correction and fixed with either three lateral 1.5 mm K-wires or two lateral and one medial K-wire. The medial K-wire was inserted through a small incision in the elbow extension, slightly anteriorly to protect the ulnar nerve. Intraoperative assessment of correction was verified clinically with a goniometer and with imaging. Layered wound closure was done, and a padded long arm back slab was applied in flexion.

Postoperative and rehabilitation protocol

Active finger movements are encouraged on the first postoperative day, and elevation of the limb is advised to reduce swelling. Initial wound inspection was done on the fourth postoperative day, and discharge was permitted if there were no signs of infection, satisfactory wound healing, and adequate pain control. Suture removal was performed 10 to 12 days postoperatively. At three weeks, gentle ROM exercises were initiated out of the splint. Four to six weeks postoperatively, the K-wire was removed and the plaster splint discontinued. This was followed up with progressive ROM exercises.

Statistical analysis

The collected data were first organized into Microsoft Excel (Microsoft Corp., Redmond, WA, USA) before being analyzed using SPSS Statistics version 20 (IBM Corp., Armonk, NY, USA). Descriptive statistics, including mean values and 95% confidence intervals, were calculated. For group comparisons, independent t-tests were used for two groups, whereas ANOVA was applied for analyses involving three or more groups. Paired t-tests were used to compare pre- and post-treatment measurements, along with regression analysis to assess potential correlations. A p-value of less than 0.05 was considered statistically significant.

## Results

The study included 18 participants with a mean age of 10.72±2.89 years. The average interval between injury and surgical intervention was 17.4 months (±5.8). The cohort was predominantly male (72.2%, n = 13), with left-side involvement more common (61.1%, n = 11) than right-side (38.9%, n = 7). Most patients (55.5%, n=10) received no formal treatment (using homemade slings or traditional bandaging). Around 27.8% (n=5) were managed non-surgically (with plaster), while only 16.7% (n=3) underwent surgery. Preoperative measurements of the unaffected side are summarized in Table 1.

**Table 1:** Table showing mean age and duration since injury and measurements of the normal side HUA: Humero-ulnar angle, LCPI: Lateral condylar prominence index

Mean ± SD	Age (years)	Duration since injury (months)	HUA on normal side	LCPI normal side	Flexion normal side	Extension normal side	Arc normal side
	10.72 ± 2.89	17.54 ± 5.8	11.72°±2.10°	10.29 ± 4.38	141.11°±3.66°	6.56° ± 3.61°	147.39°±4.32°

**Figure 5:**
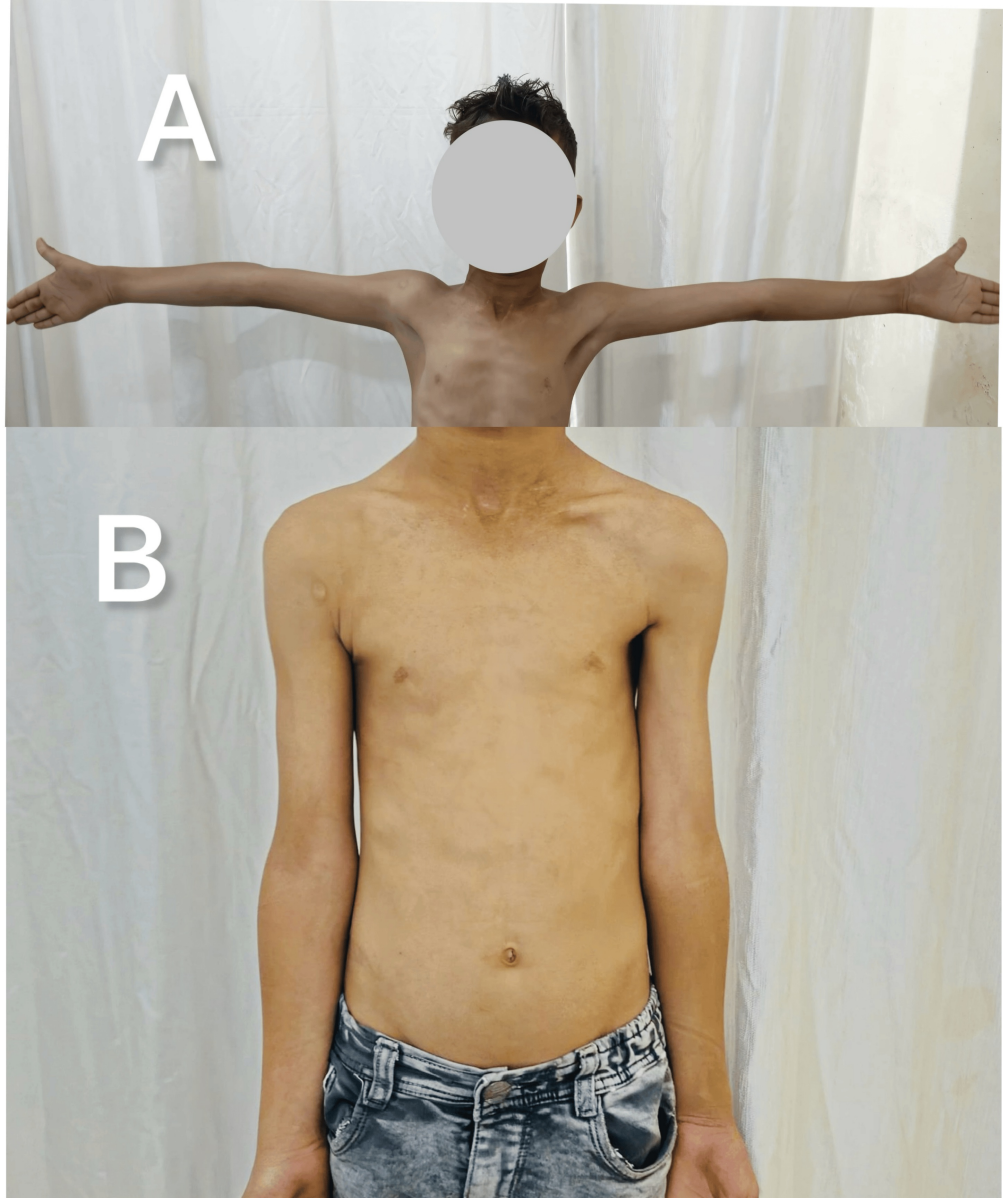
Clinical image of the same patient from Figure 1 showing correction of the varus deformity three months post the osteotomy

**Figure 6:**
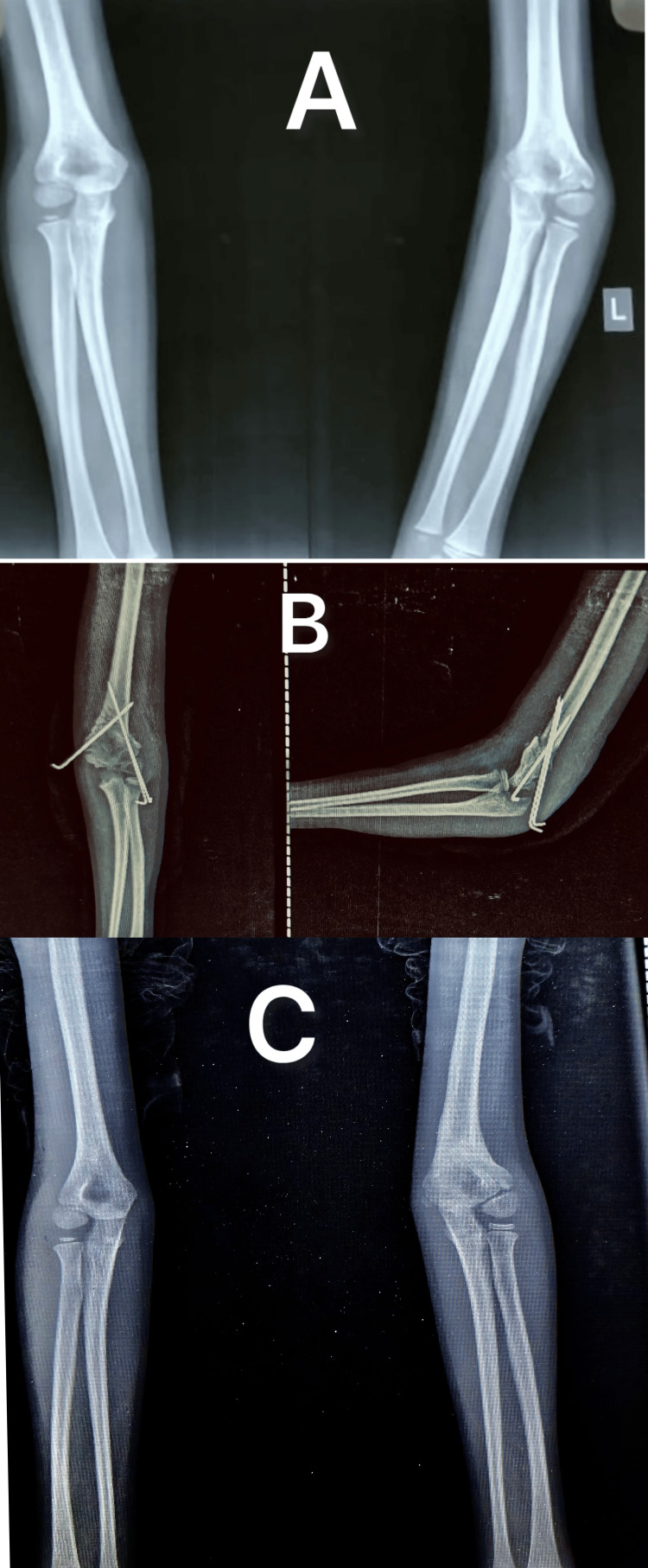
Radiographs showing cubitus varus of the left elbow (A) corrected with a dome osteotomy (B) and achieving proper alignment and healing by three months (C)

The affected side exhibited a varus deformity, with a mean HUA of -12.93° ± 2.93 and a clinical CA of -13.40° ± 2.69. Postoperatively, the HUA improved significantly to 9.11° ± 2.49 (shifting toward valgus), while the clinical CA improved to 9.94° ± 2.99, demonstrating successful correction of the deformity (Figures 5-6).

The mean elbow ROM improved significantly from 117.5° ± 11.28 in flexion and 9.3° ± 4.6 in extension to 131.9° ± 8.0 in flexion (difference=14.44, 95% CI=7.79-21.08, t-stat=4.4, P=0.0001) and 3.78° ± 3.65 in extension (difference=-5.56, 95% CI=-8.37 to -2.74, t-stat=-4.01, P=0.0003). The MEPS showed a modest increase from 90.83 ± 12.74 (preop) to 93.33 ± 9.23 (postop); however, this improvement was not statistically significant (p=0.5, difference=2.5, 95% CI: -5.03 to 10.03, t-stat=0.67). In contrast, the LCPI demonstrated a marked correction, shifting from -7.66% ± 6.56 preop to 10.13% ± 7.34 postoperatively (difference=17.79, 95% CI: 13.07 to 22.5, t-stat = 7.67, p < 0.0001) (Table 2). According to the Banerjee criteria, 83.33% (n=15) of patients achieved excellent outcomes, while 16.67% (n=3) had good outcomes, with no poor outcomes.

**Table 2:** Analysis of different outcomes measures HUA: Humero-ulnar angle, LCPI: Lateral condylar prominence index, MEPS: Mayo elbow performance score

Parameter	HUA	LCPI	Flexion	Extension	Arc	MEPS
Preoperative mean ± SD	-12.93°±2.93°	-7.66±6.56	117.5°±11.28°	9.33°±4.63°	126.83°±10.42°	90.83±12.74
Postoperative mean ± SD	9.11°±2.49°	10.13 ± 7.34	131.94°±8.06°	3.78°±3.65°	136.27°±6.68°	93.33±9.23
Difference	22.04	17.79	14.44	-5.56	9.44	2.5
95% CI	20.19 to 23.88	13.07 to 22.5	7.79 to 21.08	-8.37 to -2.74	3.51 to 15.36	-5.03 to 10.03
T-stat	24.31	7.67	4.4	-4.01	3.23	0.67
p-value	<0.0001	<0.0001	0.0001	0.0003	0.0027	0.5

Analysis of outcome correlations revealed that age at presentation showed a statistically significant positive correlation with time to union (r=0.6, p=0.003). However, age demonstrated no significant association with postoperative CA, LCPI, ROM, MEPS, or Banerjee criteria (p > 0.05). Furthermore, none of the other examined variables, including prior treatment, duration since injury, affected side, or patient gender, showed any significant correlation with the primary outcome measures (CA, LCPI, ROM, MEPS, time to union, or Banerjee criteria), with all comparisons yielding p-values > 0.05.

## Discussion

Cubitus varus deformity typically does not impair functional capacity; it presents primarily as a cosmetic concern. The primary treatment objectives involve restoration of normal anatomical alignment to match the contralateral side and prevention of potential long-term sequelae, achievable through various corrective osteotomy techniques. Among these, dome osteotomy, with its rotational axis centered within the distal humeral metaphysis, has demonstrated particular efficacy in addressing rotational deformities while simultaneously minimizing lateral condylar prominence [3,11]. Our retrospective study evaluating dome-shaped supracondylar osteotomy for post-traumatic cubitus varus revealed statistically significant improvements in postoperative CAs, ROM, LCPI, and Banerjee criteria, with no major complications observed. In the current study, the majority of patients (55.5%, n=10) received no formal medical treatment, relying instead on homemade slings or traditional bandaging administered by local practitioners (quacks), thus highlighting potential gaps in access to professional care or a preference for informal management. Just over a quarter (27.8%, n=5) were treated conservatively with immobilization like casts or slabs, and only a small fraction (16.7%, n=3) underwent surgery, indicating either low rates of severe injuries or possible barriers to surgical access. The high proportion of untreated or traditionally managed patients raises questions about healthcare accessibility, patient education, or cultural practices influencing care-seeking behavior, particularly in resource-limited settings.

Gurung et al. [15] demonstrated the efficacy of dome osteotomy in their study of 65 patients, reporting significant correction of cubitus varus deformities with CA improving from 23.90°±8.84 varus to 5.14°±7.64 valgus, LCPI changing from 0.58±16.68 to -3.18±16.39, and MEPS increasing from 89.54±6.66 to 95.23±5.26. Similarly, a retrospective study of 18 patients with severe cubitus varus (>30°) treated with shortening dome osteotomy [16] showed HUA correction from 26.1° varus to 7.3° valgus along with LCPI improvement from -2.4 to -1.7. While these studies and our current research share a retrospective design, our findings align with their reported outcomes despite differences in cohort characteristics. Our study demonstrates HUA correction from -12.93°±2.93 to 9.11°±2.49 and LCPI improvement from -7.66±6.56 to 10.13±7.34, representing comparable corrective outcomes, albeit in a smaller sample size (compared to Gurung et al. [15]), with less severe initial deformities compared to the referenced studies. Although we observed a slight improvement in the mean postoperative MEPS (93.33 ± 9.23) compared to baseline (90.83 ± 12.74), this difference was not statistically significant. The high baseline MEPS score, already within the 'excellent' range, further supports the notion that cubitus varus is primarily a cosmetic concern, with functional impairment being rare. Kumar et al. [17] similarly reported favorable outcomes using dome osteotomy for cubitus varus correction, despite their relatively small cohort of 10 patients (mean age 9.1 years). Their results demonstrated significant improvement in both angular and LCPI measurements: the CA corrected from a preoperative mean of -20.1° (versus 10.4° on the unaffected side) to a postoperative mean of 8.2°, while the LCPI decreased from a preoperative range of 2.5% to 4.5% (mean 3.5%) to a postoperative range of 2.0% to 2.7% (mean 2.3%). Notably, successful outcomes have also been demonstrated in adult populations, with a study of six patients undergoing dome osteotomy for cubitus varus achieving a mean postoperative CA of 9.72° and showing mean LCPI improvement of 6.92% [18]. These results indicate that the technique remains effective for deformity correction even in skeletally mature patients.

The functional requirements for elbow ROM in daily activities have been progressively redefined. While Morrey et al.'s classic 1981 study established 30° to 130° as the essential arc [19], contemporary research demonstrates more demanding physiological needs. Oosterwijk et al.'s 2018 systematic review of 36 studies encompassing 66 activities of daily living revealed that unimpaired individuals regularly utilize full elbow flexion (150°) during personal care and feeding tasks [20]. This finding is corroborated by Raiss et al.'s detailed 3D motion analysis, which documented a functional range of 0° to 146° (mean arc 110°) across 10 standardized activities, including grooming, eating, and hygiene activities in healthy young adults [21]. One of the factors that might influence postoperative mobility outcomes is the surgical approach. Our study cohort, with a mean age of 10.72±2.89 years and treated via a paratricipital approach, achieved superior postoperative ROM (mean arc 136.27°±6.68, range 125° to 150°) compared to published results using triceps-splitting techniques. Gurung et al. reported a more limited postoperative arc (<100°, range 120-129.5°) in their series [15], while Kumar et al. documented outcomes of 100° to 120° [17]. These differences likely reflect both technical factors and population characteristics, as our younger patient cohort may have had better preserved mobility or overall greater ROM. In addition, multiple other factors may contribute to ultimate ROM outcomes following cubitus varus correction. While the surgical approach (triceps-sparing versus splitting) remains significant, preoperative ROM, age, and postoperative rehabilitation are equally important determinants. The consistently good functional outcomes across all studies, despite varying postoperative ROM measurements, suggest that compensatory mechanisms and individualized rehabilitation may mitigate motion limitations. Our findings particularly highlight the potential advantages of the paratricipital approach in pediatric populations, though the multifactorial nature of postoperative recovery warrants comprehensive preoperative assessment and tailored postoperative management.

The reported complications of corrective osteotomies for cubitus varus include infection, nerve injuries (predominantly ulnar nerve), loss of reduction, decreased range of motion, and unsatisfactory scarring [6,7,15,22,23]. A systematic review of surgical techniques for cubitus varus correction by Hoffman et al. [6] reported an infection rate of 9.45% in patients treated with dome osteotomy and 4.7% of patients experiencing loss of motion. In another study [15], nerve injuries occurred in 6.15% of cases (three involving the ulnar nerve and one the radial nerve), with three cases recovering fully by final follow-up. Superficial skin infections were reported in 3.08% of patients, all resolving with standard wound care. Kumar et al.'s study [17] documented similar complications, including one case of ulnar neuropraxia, three pin tract infections, and two superficial skin infections. In our series, we observed ulnar nerve neuropraxia in two patients where medial pins were employed, both achieving complete recovery within 10 weeks. Two additional cases developed minor wound gaping, which healed satisfactorily with conservative management within one week. One patient experienced postoperative osteotomy displacement requiring revision with plate fixation. While conventional dome osteotomy remains a reliable and reproducible technique for severe deformity correction, it creates a significant valgus moment that may potentially stretch neurovascular structures. This has led to the development of shortening dome osteotomy, which maintains the advantages of the conventional approach while reducing tension on neurovascular bundles [24]. Notably, our study did not encounter any neurovascular complications related to excessive tension, suggesting that with proper technique, conventional dome osteotomy can be performed safely without these specific concerns. Intraoperatively, the major challenge we faced was difficulty in rotating the distal fragment to achieve optimal correction, particularly in patients with larger deformities, and the inability to accurately assess rotational alignment.

This study has several limitations. The retrospective nature may introduce biases, including selection bias and inconsistent documentation. The absence of a control group hinders comparative analysis with alternative treatments. Further limitations include variability in preoperative management, a single-center cohort, and a small sample size, potentially limiting generalizability. Additionally, rotational deformity, a critical factor in cubitus varus, was not assessed. Interobserver variability may also arise from multiple clinicians recording pre-treatment data.

## Conclusions

Dome osteotomy demonstrates consistent safety and efficacy in correcting post-traumatic cubitus varus in children, with minimal complications, as objectively measured by LCPI, MEPS, and the Banerjee criteria. Future multicenter studies should leverage advanced 3D imaging and motion analysis to evaluate long-term skeletal remodeling patterns, compare biomechanical outcomes of different osteotomy techniques, and validate standardized radiographic parameters for surgical planning. These evidence-based advancements will optimize age-specific treatment algorithms and improve decision-making in pediatric orthopedic practice.
